# Successful Downstaging of High Rectal and Recto-Sigmoid Cancer by Neo-Adjuvant Chemo-Radiotherapy

**DOI:** 10.4137/cmo.s348

**Published:** 2008-03-01

**Authors:** Brian O’Neill, Gina Brown, Andrew Wotherspoon, Sarah Burton, Andy Norman, Diana Tait

**Affiliations:** 1 Department of Clinical Oncology, Mayday University Hospital, Croydon, U.K; 2 Department of Radiology, Mayday University Hospital, Croydon, U.K; 3 Department of Histopathology, Mayday University Hospital, Croydon, U.K; 4 Division of Colorectal Surgery, Mayday University Hospital, Croydon, U.K; 5 Medical Statistics, Royal Marsden Hospital, Sutton, Surrey, U.K

**Keywords:** chemoradiotherapy, chemotherapy, radiotherapy, total mesorectal excision, downstaging

## Abstract

**Purpose:**

The benefit of neoadjuvant therapy for tumours above the peritoneal reflection is not clear. The purpose of this study is to demonstrate the feasibility and downstaging of treating locally advanced tumours from high rectum to distal sigmoid with preoperative chemoradiotherapy (CRT).

**Methods and Materials:**

Seventeen patients with high rectal, rectosigmoid or distal sigmoid tumours above the peritoneal reflection received neoadjuvant CRT, selected on MRI findings indicating T4 disease or threatened circumferential resection margin. All patients were administered neoadjuvant chemotherapy, with Oxaliplatin or Mitomycin C and a Fluoropyrimidine. The pelvis received long-course CT-planned conformal RT, 45 Gy in 25 fractions, with a boost of 5.4–9 Gy in 3–5 fractions. Thirteen patients were treated with concomitant oral or intravenous Fluoropyrimidine chemotherapy.

**Results:**

Median follow-up was 37 months. Overall survival was 82.35% (95% Confidence Interval (CI) 54.7–93.9) and disease free survival 81.25% (95% CI 52.5–93.5). Only 1 patient suffered loco-regional relapse. Chemotherapy regimens were well tolerated, though some patients required dose reductions. Nine patients (52.9%) lowered pathologic disease AJCC stage, i.e. ‘downstaged’. Six patients (35.3%) achieved complete pathological response. Clear margins were attained in all but 1 patient. Three patients were converted from cT4 to ypT3. No patient required a gap during CRT. One patient suffered a grade III acute toxicity, but no grade IV (RTOG). There were 3 grade III and 3 grade IV late toxicities (LENT-SOMA).

**Conclusions:**

Locally advanced high rectal and recto-sigmoid tumours may be treated with pre-operative CRT with acceptable toxicity, impressive down-staging, and clear surgical margins.

## Introduction

The position of tumours within the rectum, in terms of their height above the anal verge, influences the likelihood of local control and also the impact that radiotherapy has on achieving local control. Low tumours requiring abdomino-perineal resection (APR) carry a much higher local recurrence rate compared with those tumours that are treated by anterior resection (Marr et al. 2005). This at any rate applies when a conventional APR technique is employed which, by following normal anatomy contours, means that the resection margin is very close to tumour in the region of the origin of the levators. As a result, there is a far higher rate of histopathological margin positivity in the resected specimen following APR. For these low tumours, neoadjuvant radiotherapy reduces local failures in operable tumours and provides useful down-staging in locally advanced disease (Sauer et al. 2004).

For tumours higher in the rectum, and those at the recto-sigmoid junction, the benefit of neoadjuvant therapy is not as clear. In the Dutch Colorectal Cancer Group Trial, randomising patients undergoing total mesorectal excision to neoadjuvant short course radiotherapy, there was no significant benefit in terms of local control in tumours situated 10.1–15 cms from the anal verge (Kapiteijn et al. 2001). This was in contrast to tumours located lower in the rectum with both tumours arising between 5 and 10 cms, and those below 5 cms from the anal verge, showing a significantly reduced local recurrence rate. This is an intuitive result with radiotherapy being able to provide a benefit in the location which is most likely to be associated with positive surgical resection margins. However, although it’s easy to define the location of very low tumours, because of their accessibility to the examining finger and easier measurement of distance from the anal verge on imaging, it becomes increasingly difficult to determine the position of tumours higher in the rectum and in establishing the position of the recto-sigmoid junction. The variability of measurement on sigmoidoscopy is well recognised and the division of the rectum by strict centimetre criteria is unrealistic on an individual basis. The lack of clarity in agreement in the available randomised data as to how tumours are designated as rectal, as opposed to sigmoid, means that high rectal, rectosigmoid and even distal sigmoid cancers are sometimes included in rectal cancers series. A useful anatomical marker is the peritoneal reflection which prior to MRI could not be reliably located, except at surgery. The peritoneal reflection is a critical marker as the tissues inferior to this structure form a discreet compartment which can be encompassed within a radiotherapy target volume, whereas tumour above this structure is considered likely to disseminate within the peritoneal cavity and be beyond the scope of a useful and tolerable radiotherapy volumes (Pilipshen et al. 1984). For this reason, there is a generally held perception that neoadjuvant radiotherapy is not appropriate for high rectal and rectosigmoid tumours, having a greater potential for acute and chronic side-effects, due to the proximity of small bowel, without the potential level of efficacy achieved with tumours in the low to mid rectum. Consequently, radiation oncologists can be biased against the application of neoadjuvant or adjuvant radiotherapy for these tumours.

For rectal cancer, it is now appreciated that the margin of excision and the risk of a positive circumferential resection margin (CRM) can be accurately predicted by high spatial resolution pre-operative MRI (MERCURY, 2006). Recently, it has been shown that this ability to accurately identify and stage tumours pre-operatively by MRI can be extended to tumours in the upper rectum, rectosigmoid and distal sigmoid (Burton et al. 2006). A group of patients with MRI defined threatened CRM and T4 tumours situated either in the high rectum, recto-sigmoid or distal sigmoid have been treated in our institution with a neoadjuvant CRT schedule with the aim of down-staging prior to surgery. Such a group has not been previously identified in any of the trials using CRT in locally advanced disease. In this article the feasibility of applying such a strategy to these high tumours is demonstrated and the subsequent down-staging, 3 year local failure rate and survival is presented.

## Methods and Materials

### Study population

Seventeen patients with high rectal, recto-sigmoid or distal sigmoid received neo-adjuvant CRT in an attempt to downstage their tumours pre-operatively. They were selected on the basis of MRI findings which indicated either a T4 tumour or T3 tumour with a ‘threatened’ CRM, and discussed at the centre Multi-Disciplinary meeting. A threatened CRM is defined as tumour or nodal disease on MRI at or within 1 mm of the mesorectal fascia, the boundary of dissection in total mesorectal excision (TME). All tumours were located above the peritoneal reflection. Based on MRI assessment, the lower border for upper rectal tumours was defined as 10 cm above the anal verge, for recto-sigmoid tumours 12 cm, and for distal sigmoid tumours 15 cm.

### Pre-operative MRI technical details

Pre-operative supine T2 MR scanning was performed on a 1.5T Siemens scanner with a 4-element pelvic phased array wrap-around surface coil. Intravenous contrast was not used. All scans were reviewed by a single radiologist (GB).

### Radiotherapy technical details

**Figure 1:**
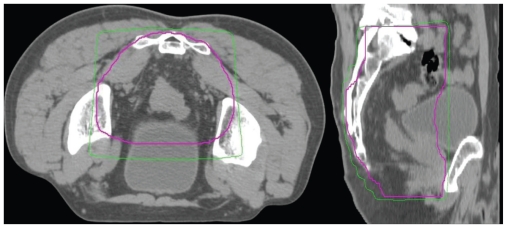
Axial and Sagittal phase I planning CT showing planning target volume (thick line) and 95% isodose (thin line). Note a higher than standard superior border to cover primary disease with a 3 cm margin.

All patients received megavoltage radiation (6 or 10 MV) via 2-phase CT-planned conformal RT (see Fig. 1). A planning CT scan was performed in the treatment position, with an anal marker. Patients were treated prone with a full bladder. A 3 or 4 field technique was used for both phases, with segmented low-weighted fields to reduce ‘hot-spots’ where necessary. Conformal blocks or multi-leaf collimators (MLC) were used on all fields. Treatment planning was performed with computerized dosimetry and the prescribed dose specified according to ICRU 50 guidelines (1993). All fields were treated each day, 5 days a week. Electronic Portal Imaging of each field was performed daily for the first 4 fractions. Further imaging was not routinely performed once images were within a 5 mm tolerance in all planes.

**Figure 2:**
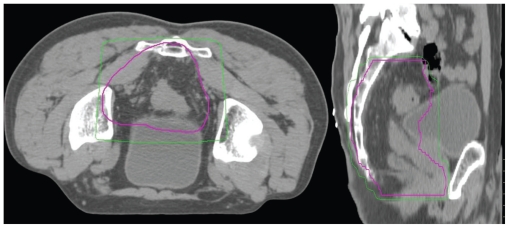
Axial and Sagittal phase II planning CT showing planning target volume (thick line) and 95% isodose (thin line). Note a 2 cm margin around the gross tumour volume (including nodal disease in the mesorectum), with anterior sacrum included.

Departmental policy for pre-operative RT for rectal cancer is to treat the pelvis with long-course RT, 45 Gy in 25 fractions at 1.8 Gy per fraction, with concomitant chemotherapy. A boost of 5.4–9 Gy (the lower dose if there is a concern about the volume of small bowel included in the boost field) in 3–5 fractions at 1.8 Gy per fraction is administered (see Fig. 2).

For the phase I treatment, the clinical target volume included the rectal tumour with a margin, the mesentery, and perirectal, presacral, internal iliac, external iliac and distal common iliac lymph nodes. The anterior margin was increased to include external iliac nodes if the primary tumour invaded bladder, prostate, cervix or vagina. The posterior margin included the sacrum. Lateral margins included the pelvic side-wall nodes. Superior margins are in the region the L5/S1 interspace, though for high rectal tumours, this margin may be increased to allow a 3 cm margin superior to the extent of the primary tumour, as identified on pre-operative MRI. The inferior border depends upon the level of tumour within the rectum. For low rectal tumours within 3 cm of the anal verge the perineum is included, but where possible and allowing a 3cm margin below the inferior extent of the tumour, the muscles of the anal sphincter are spared.

The phase II volume covers assessable tumour (via MRI, CT, and clinical examination) with a 2 cm margin.

### Follow-up and assessment of toxicity

Patients were reviewed weekly during RT to assess toxicity. Those in a formal trial had detailed toxicity documentation as per trial protocol, while those treated outside of a trial setting had toxicity recorded according to departmental protocol. Four weeks after completion of CRT, acute toxicity was re-assessed with surgery following approximately two weeks later. Late toxicity was assessed at each subsequent follow-up visit. After surgery, patients were seen in clinic at three-monthly intervals for the first year, six-monthly for the next two years, and then yearly until five years had elapsed post-operatively. Clinical examination, digital rectal examination where appropriate, and tumour marker (Carcinoembryonic Antigen) were assessed at each visit. A CT thorax, abdomen, pelvis was routinely performed at one and two years postoperatively. Clinical or radiological suspicion of local recurrence necessitated MRI pelvis for anatomical definition, and pathological confirmation.

### Histopathology

All resections were performed with curative intent. Histopathologic assessment was performed according to Royal College of Pathologists guidelines (Quirke P, 1998). In cases of apparent histopathological complete response, multiple blocks throughout the entire rectum were obtained.

### Chemotherapy

Details of neo-adjuvant, concomitant and adjuvant chemotherapy are summarized in Table 1. Departmental policy for concomitant chemotherapy is oral Capecitabine 825 mg/m^2^ twice daily, including weekends.

### Statistical analysis

Survival was analysed using the Kaplan-Meier method. 95% confidence intervals were calculated using the binomial distribution.

## Results

### Patient characteristics

Of the 17 patients reviewed, 9 were male and 8 female. Median age at diagnosis was 61 years (range 27–79). By the radiological criteria outlined above, 8 (47%) patients had tumours in the upper rectum, 5 (29.4%) in the recto-sigmoid, and 4 (23.5%) in the distal sigmoid. Eight patients had been recruited into the ‘EXPERT’ trial, a phase II study of neo-adjuvant Oxaliplatin and Capecitabine and pre-operative CRT with concomitant Capecitabine, for patients with locally advanced and inoperable rectal cancer (Chau et al. 2006). Four further patients were treated off-study according to the ‘EXPERT’ protocol. Three patients were treated off-study according to the protocol of an earlier trial at the Royal Marsden of 12 weeks of neo-adjuvant protracted infusional 5-Flourouracil and Mitomycin-C both prior to CRT and 12 weeks post-operatively (Chau et al. 2003). One patient was recruited into a phase II trial of Capecitabine and Mitomycin-C as initial treatment for patients with metastatic colorectal cancer (Rao et al. 2004).

### Treatment outcome

Median follow-up is 37 months (range 32–64 months). Overall survival is 82.35% and disease free survival 70.59%. Loco-regional relapse rate is 5.88% (1 patient, pre-sacral recurrence). Two further patients, both of whom have died, had peritoneal disease, which was interpreted as meta-static. All end-points were calculated from the first day of neo-adjuvant therapy. We did not attempt a univariate analysis of tumour position due to small numbers.

### Radiotherapy

All patients received long-course RT as described. Fifteen patients received 54 Gy in 30 fractions in 2 phases, 2 patients 50.4 in 28 fractions. There were no gaps in treatment as a result of toxicity.

### Chemotherapy

All patients received neo-adjuvant chemotherapy. Twelve patients were administered 4 cycles of neo-adjuvant Oxaliplatin/Capecitabine within the ‘EXPERT’ protocol or with the same protocol ‘off-trial’. Seven completed 4 cycles as per protocol. Of the remaining 5, only 1 patient required treatment cessation, the remainder completing 4 cycles with dose reductions. Four patients tolerated neo-adjuvant Mitomycin-C based regimens well, with only 1 patient requiring a minor 5-Flourouracil dose-reduction.

Thirteen patients tolerated concomitant chemotherapy well. Only 2 patients required a 25% dose reductions of Capecitabine. Eight patients completed adjuvant chemotherapy as per protocol. One patient discontinued adjuvant therapy due to development of pulmonary metastases. Two patients had dose reductions for grade III diarrhoea, 1 for grade IV diarrhoea, and 1 for grade III stomatitis.

### Pathology

**Figure 3:**
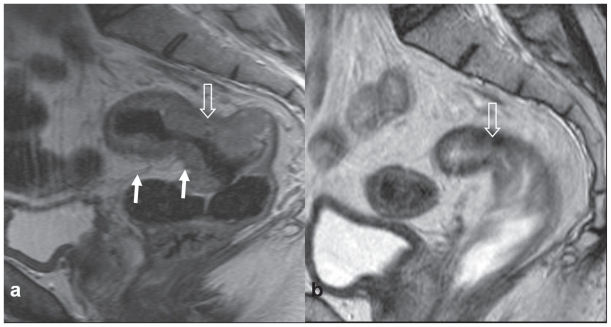
Patient 1: Sagittal T2 weighted fast spin echo (TR3900 TE 120) MRI scans of **a**) pre-chemoradiotherapy, showing a large rectal tumour (open arrow) almost entirely above the peritoneal reflection (arrow), and **b**) marked tumour regression and downsizing 4 weeks post-chemoradiotherapy, with areas of low signal intensity indicating fibrosis (open arrow).

**Figure 4:**
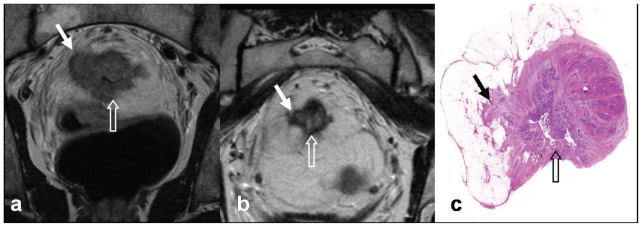
Patient 1: Coronal oblique imaging axial to Recto—Sigmoid, T2 weighted fast spin echo (TR4000 TE 120) MRI scans demonstrating: evidence of Recto-Sigmoid adenocarcinoma downstaging with neoadjuvant chemoradiotherapy. **a**) Annular tumour invading extensively into the mesorectum, with a threatened circumferential surgical margin (arrow) and tumour also appears to have perforated through the peritoneal reflection (open arrow). Preoperative chemoradiotherapy was administered. **b**) Scans 4 weeks following completion of therapy. There has been remarkable tumour regression, with low signal intensity indicating fibrosis (arrow) and margins no longer appear threatened. There is fibrosis (low signal intensity) at the peritoneal reflection. **c**) Surgical specimen confirms fibrosis (arrows). This was an R0 resection.

**Figure 5:**
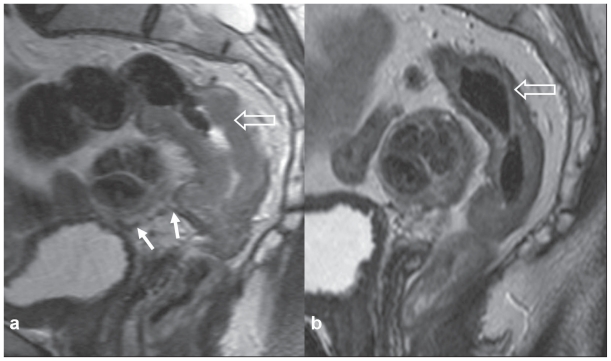
Patient 2: Sagittal T2 weighted fast spin echo (TR3900 TE 120) MRI scans of **a**) pre-chemoradiotherapy, showing a large rectal tumour (open arrow) arising entirely above the peritoneal reflection (arrowed) into the sigmoid colon, almost to the level of S1/2, and **b**) considerable tumour regression and downsizing 4 weeks post-chemoradiotherapy, with extensive low signal intensity indicating fibrosis (open arrow), consistent with a radiological complete response.

**Figure 6:**
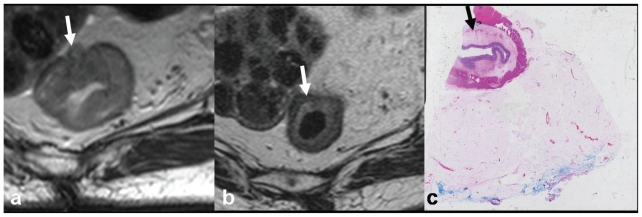
Patient 2: Oblique axial T2 weighted fast spin echo (TR4000 TE 120) MRI scans demonstrating evidence of Recto-Sigmoid adenocarcinoma downstaging with neoadjuvant chemoradiotherapy. **a**) Near circumferential tumour abuts (arrowed) the peritoneal surface and fibroid uterus. Preoperative chemoradiotherapy was delivered. **b**) Scans 4 weeks following completion of therapy. There has been very considerable tumour regression, with extensive low signal intensity indicating fibrosis (arrowed). Appearances are in keeping with a radiological complete response. **c**) Surgical specimen confirms fibrosis (arrowed) and was a pathological complete response.

Clinical, and corresponding Pathological stages, are represented in Table 2. All patients had threatened surgical resection margins by MRI criteria. For T3 tumours, this margin could be threatened by tumour or nodal disease within 1 mm or at the mesorectal fascia, or a surgical margin could be threatened by virtue of T4 disease. All patients underwent TME. Three patients had cT4 disease, while 1 could not be distinguished between T_3b_ and T_4_. One patient with a cT4N2M0 tumour infiltrating towards the left pelvic side wall and encasing external iliac vessels had a positive pathological circumferential resection margin (ypT3N1M0). All others underwent R0 resection. On initial pre-treatment MRI, 2 patients had pelvic side wall nodes, 1 had an inferior mesenteric node, 2 had tumour directly invading the pelvic side wall, and 1 a nodule near the sacrum on mesorectal fascia. Nine patients (52.9%) achieved a lower pathologic disease AJCC stage (2002) following neo-adjuvant therapy and surgery, i.e. were ‘downstaged’. This is demonstrated by Patient 1, sagittal (Fig. 3a and 3b) and axial MR views (Fig. 4a and 4b), with corresponding histopathology (Fig. 4c). Six patients (35.3%) achieved a complete pathological response despite 2 of these being cT4 and a third a borderline T4. Complete pathological response is demonstrated by Patient 2, sagittal (Fig. 5a and 5b) and axial MR views (Fig. 6a and 6b) of the same patient, with corresponding histopathology (Fig. 6c).Three patients were converted from cT4 to ypT3. Two patients assigned cN2 on pre-treatment MRI proved ypN0.

### Acute and late toxicity

Acute and late toxicities are shown in Table 3. Acute toxicity is demonstrated using the acute RTOG scoring system (acute cutaneous toxicity was not included). Only 1 patient suffered a grade III toxicity, while there were no grade IV acute toxicities. No patient required a gap in treatment during CRT.

Late toxicity is presented with the Lent**-**Soma system. There were 3 grade III and 3 grade IV late toxicities.

## Discussion

This series was comprised of patients with high-risk locally advanced tumours situated either high in the rectum, the recto-sigmoid or distal sigmoid. All were at risk of involved surgical margins by pre-operative radiological criteria. In spite of this, more than a third (35.3%) of patients achieved a pathological complete response, and clear margins were attained in all but one patient. More than half of the patients (52.9%) had their tumours ‘down-staged’ and CRT was delivered with minimal early and late side-effects, achieving a 3-year survival of 82.35% (95% CI 54.7–93.9), and a 3-year disease-free survival of 81.25% (95% CI 52.5–93.5). Long term results are awaited, though it is anticipated that such a degree of down-staging will be associated with a favorable long term outcome (Rodel et al. 2005; Mawdsley et al. 2005; Balch et al. 2003; Shivnani et al. 2004). Our data therefore suggests that such high-risk locally advanced high rectal and recto-sigmoid tumours may be treated with pre-operative CRT with acceptable toxicity and impressive down-staging, rendering clear surgical margins in most patients, with an excellent short term outcome.

All patients underwent optimal surgery with TME, as described by Heald (Heald and Ryall, 1986). CRM status has emerged as one of the most important prognostic determinants following TME (Mawdsley et al. 2005). The ability of pre-operative MRI to accurately predict CRM status has been validated recently in a multi-centre European trial (MERCURY, 2006).

All of the patients in this series received neo-adjuvant chemotherapy prior to concomitant CRT. It is now established that this approach for locally advanced rectal cancer results in excellent down-staging, pCR rates and margin negativity (Chau et al. 2006). In the EXPERT study patients with MRI—defined poor risk features were treated with 4 cycles of neoadjuvant Oxaliplatin and Capecitabine prior to radiotherapy with concomitant Capecitabine. The schedule was well tolerated, and 87% of patients proceeded to surgery 6 weeks after completing radiotherapy. The pathological complete response rate was 24% and specimens with only microscopic residual tumour foci accounted for a further 48%. Because of the effectiveness of this schedule in tumours with a threatened circumferential margin within the rectum, the same schedule was applied to those patients with higher tumours. An additional imperative for the addition of neoadjuvant chemotherapy may be the early treatment of micro-metastatic disease with the aim of reducing the rate of subsequent systemic relapse. Confining chemotherapy to that given concomitantly with radiotherapy has not had an impact in overall survival in a randomised trial of preoperative RT versus CRT in T3–4 Rectal Cancers, despite the addition of this concomitant chemotherapy producing superior downstaging (Gerard JP et al. 2005). This implies that concomitant chemotherapy provides effective radio-sensitisation but is not tumoricidal, whether by choice of agent or dose, in terms of eradicating micro-metastases. It will be fundamental to the testing of the EXPERT schedule that a reduction in metastatic disease and superior disease free and overall survival will result from inclusion of neoadjuvant chemotherapy. Furthermore, although there may be a sound basis for giving this extent of chemotherapy neo-adjuvantly, it must permit tolerable delivery of the other contributing treatment modalities, surgery and radiotherapy.

No fixed bony landmark exists to distinguish the recto-sigmoid junction, nor is the distance from the anal margin a reliable or reproducible surrogate. The key pre-operative RT trials did not have the benefit of MRI, and therefore used sigmoidoscopic distance from the anal verge. The definition for upper border of ‘rectum’ has not been consistent between trials, and thus varying degrees of distal sigmoid and recto-sigmoid tumours have been included. The German Rectal Cancer Study Group accepted all tumours with an inferior border within 16 cm of anal verge (Sauer et al. 2004); tumours in the Swedish Rectal Cancer Trial had to be below the sacral promontory on a lateral barium enema (1997). The Dutch Colorectal Cancer Group used an amalgamation of these policies, an inferior border not further than 15 cm from the anal verge and below the level of S1–2 (Kapiteijn et al. 2001). This strongly implies that significant numbers of distal sigmoid and recto-sigmoid tumours were included in these trials.

In the Dutch trial, location was a significant predictor of local recurrence. RT had no effect on local recurrence for tumours between 10 and 15 cm from the anal verge, with a hazard ratio of 1.00 (p = 0.17). This result supports the prejudice against neo-adjuvant RT for treating higher rectal cancers.

The Swedish group did not perform a univariate analysis to review the relative local control benefit depending on the location of tumour within the rectum (1997). Nor have the two major met-analyses of pre-operative and post-operative RT investigated this (Camma et al. 2000 2001). Only one of the adjuvant RT trials after 1990, used in these meta-analyses, has performed a univariate analysis of different anatomical parts of the rectum and there was no demonstrable impact on outcome (Treurniet-Donker et al. 1991).

These trials therefore appear to have routinely included distal sigmoid and recto-sigmoid tumours, which may not benefit from ‘routine’ pre-operative radiotherapy. The rationale for irradiating tumours above the peritoneal reflection is far less well established than is the case for rectal tumours. These high tumours generally fail in a colonic pattern with peritoneal, rather than local failure, though high local recurrence rates have been reported, and this would support the application of a neo-adjuvant strategy, similar to the approach to rectal cancer (Pilipshen et al. 1984).

To our knowledge, no series of neo-adjuvant CRT exists for sigmoid or colonic tumours. Willett et al. at the Massachusetts General Hospital published the largest retrospective series of adjuvant RT for colonic tumours (n = 203) (Willett et al. 1984). This included patients with colonic T4N0M0 tumours, T3N1-2 in anatomically ‘immobile’ regions (i.e. retroperitoneal), and selected high risk T3N0 tumours with close margins. Patients were treated with adjuvant RT (45 Gy with a 5 cm margin), without concomitant chemotherapy. Treated patients were compared to a historical group (n = 395) treated with surgery only. There was a local control and disease-free survival (DFS) advantage for T4N0M0 (DFS 80%), and T4N+M0 (DFS 53%) tumours, and for T3N0 tumours with a perforation or fistula. There was a 37% 5-yr DFS in those having RT following R2 resection (i.e. gross disease left in-situ). Ten year results confirm these outcomes, especially in the T4N0 subset (Willett et al. 1993).

Intergroup-0130 was developed based on the Willett’s data but, unfortunately, this trial accrued poorly and was closed prematurely (Martenson et al. 2004). The trial design was a randomisation to test the effect of adding radiotherapy to adjuvant chemotherapy. The available data indicates that toxicity was acceptable although there was no overall survival or disease free survival advantage.

While there is a reasonable rationale to treat certain high—risk colonic tumours with adjuvant therapy, the evidence-base supporting either RT or CRT for sigmoid and colonic tumours is less robust than for rectal cancers, and there is no body of neo-adjuvant evidence. It is also apparent that randomised trials to date have included distal sigmoid and recto-sigmoid tumours, which may not benefit from ‘routine’ pre-operative RT or CRT.

## Conclusion

High rectal, recto-sigmoid and distal sigmoid tumours have not been accurately identified and reported in a trial to date. This series suggests that those that are T4 or have a threatened circumferential resection margin may be treated with pre-operative Chemo-Radiotherapy with acceptable toxicity and excellent downstaging, facilitating R0 resection and favorable short term outcome.

## Figures and Tables

**Table 1:** Neo-adjuvant, concomitant, and adjuvant chemotherapy.

Regime	Number	Dose	Comment
**Neo-Adjuvant**			
Oxaliplatin-based	12	**Oxaliplatin** 130 mg/m^2^ IV once every 3 weeks; **Capecitabine** 1000 mg/m^2^ BD PO × 14 days, 7 days rest	7 × 4 cycles app; 1 required treatment cessation, 4 completed 4 cycles with dose reductions
MMC-based	4	**MMC** 7 mg/m^2^ IV bolus every 6 weeks; -and either- **5-FU** IVI 300 mg/m^2^ /day × 12 weeks **-or- Capecitabine** 1250 mg/m^2^ BD PO x 14 days every 21 days	3 Completed app; 1 × minor 5-FU dose- reduction.
5-FU alone	1	Weekly bolus 400 mg/m^2^ × 5	Completed app
**Concomitant**			
Capecitabine	10	825 mg/m^2^ BD PO	8 Completed app; 2 × 25% dose reductions
5-FU	3	IVI 200 mg/m2/day -OR- 300 mg/m^2^ weekly bolus	2 Completed app; 1 dose reduction gr IV diarrhoea
**Adjuvant**			
Oxaliplatin/Capecitabine	2	(as neo-adjuvant)	2 Completed app
MMC-based	2	(as neo-adjuvant)	2 Completed app
Capecitabine	6	825 mg/m^2^ BD PO for 14 days, 7 days rest	2 Completed app; 3 × 25%dose reductions;1 discontinued (metastases)
5-FU	2	IVI 200 mg/m^2^ weekly for 6 weeks (i.e. 50% dose reduction); IVI 400 mg/m^2^/day weekly for 12 weeks	1 dose reduction gr IV diarrhoea; 1 Completed app

**Abbreviations:** IV: Intravenous; PO: per oral (by mouth); app: as per protocol; MMC: Mitomycin-C; IVI: Intravenous infusion; 5-FU: 5-Fluorouracil.

**Table 2:** Clinical and corresponding Pathological stages.

Clinical TNM	Clinical stage	Pathological TNM	Pathological stage	Downstaged
**cT3bN1M0**	**IIIb**	**ypT0N0M0**	**pCR**	**Yes**
cT3bNxM0	?	ypT3N2M0	IIIc	No
**cT3cN1M0**	**IIIb**	**ypT0N0M0**	**pCR**	**Yes**
cT3cN1M0	IIIb	ypT3N1M0	IIIb	No
**cT3cN2M0**	**IIIc**	**ypT3N0M0**	**IIb**	**Yes**
cT3dN1M0	IIIb	distal ypT3N0M0, proximal ypT1N0M0	IIb	Yes
**cT3dN2M0**	**IIIc**	**ypT0N0M0**	**pCR**	**Yes**
cT4N0M0	IIb	ypT3N1M0	IIIb	No
**cT4N0M0**	**IIb**	**ypT4N0M0**	**IIb**	**No**
cT4N0M0	IIb	ypT3N0M0	IIb	No
**cT4N1M0**	**IIIb**	**ypT0N0M0**	**pCR**	**Yes**
cT4N1M0	IIIb	ypT3N1M0	IIIb	No
**cT4N2M0**	**IIIc**	**ypT3N1M0 CRM +ve**	**IIIb**	**No**
cT4N2M0	IIIc	ypT0N0M0	pCR	Yes
**cT4N2M0**	**IIIc**	**ypT4N0M0**	**IIb**	**Yes**
cT_3b_/T_4_N1M0	IIIb	ypT0N0M0	pCR	Yes
**cT3cN2M1**	**IV**	**ypT3N1M1**	**IV**	**No**

**Abbreviations:** pCR: pathological complete response (no stage currently describes this).

**Table 3:** Acute (RTOG) and Chronic (LENT-SOMA) toxicities.

Toxicity	Grade I	Grade II	Grade III	Grade IV
***EARLY***				
**Bowel**	5	6	1	0
**Bladder**	6	3	0	0
***LATE***				
**Small Bowel**				
frequency	6	5	0	1
consistency	4	5	2	0
pain	1	2	0	0
**Bladder**				
frequency	1	0	1	0
Decreased stream	0	1	0	0
**Rectum**				
frequency	4	4	0	1
pain	0	2	0	0
sphincter	4	2	2	0
mucus	2	1	0	0
tenesmus	0	0	0	1

The highest late toxicity experienced is displayed, e.g. 4 patients experienced grade I urinary frequency, and no higher.
